# Challenges for Clinical Development of Vaccines for Prevention of Hospital-Acquired Bacterial Infections

**DOI:** 10.3389/fimmu.2020.01755

**Published:** 2020-08-05

**Authors:** Isabelle Bekeredjian-Ding

**Affiliations:** ^1^Division of Microbiology, Langen, Germany; ^2^Institute for Medical Microbiology, Immunology and Parasitology, University Hospital Bonn, Bonn, Germany

**Keywords:** nosocomial infection, vaccine, bacterial, colonization, immune, antibiotic resistance

## Abstract

Increasing antibiotic resistance in bacteria causing endogenous infections has entailed a need for innovative approaches to therapy and prophylaxis of these infections and raised a new interest in vaccines for prevention of colonization and infection by typically antibiotic resistant pathogens. Nevertheless, there has been a long history of failures in late stage clinical development of this type of vaccines, which remains not fully understood. This article provides an overview on present and past vaccine developments targeting nosocomial bacterial pathogens; it further highlights the specific challenges associated with demonstrating clinical efficacy of these vaccines and the facts to be considered in future study designs. Notably, these vaccines are mainly applied to subjects with preexistent immunity to the target pathogen, transient or chronic immunosuppression and ill-defined microbiome status. Unpredictable attack rates and changing epidemiology as well as highly variable genetic and immunological strain characteristics complicate the development. In views of the clinical need, re-thinking of the study designs and expectations seems warranted: first of all, vaccine development needs to be footed on a clear rationale for choosing the immunological mechanism of action and the optimal time point for vaccination, e.g., (1) prevention (or reduction) of colonization vs. prevention of infection and (2) boosting of a preexistent immune response vs. altering the quality of the immune response. Furthermore, there are different, probably redundant, immunological and microbiological defense mechanisms that provide protection from infection. Their interplay is not well-understood but as a consequence their effect might superimpose vaccine-mediated resolution of infection and lead to failure to demonstrate efficacy. This implies that improved characterization of patient subpopulations within the trial population should be obtained by pro- and retrospective analyses of trial data on subject level. Statistical and systems biology approaches could help to define immune and microbiological biomarkers that discern populations that benefit from vaccination from those where vaccines might not be effective.

## Introduction

The increasing inefficacy of antibiotics due to antibiotic resistance of bacterial pathogens has triggered the desperate search for alternative therapies. While the discovery of new antibiotics is frequently halted by concerns about toxicity or metabolism or insufficient bioavailability and tissue penetration (1, 2), the development of phage therapies has been limited by concerns about the narrow host spectrum, which requires sophisticated susceptibility testing, and the induction of neutralizing antibodies upon repeated use (3). Obviously, this situation has raised a new interest to explore the potential of vaccines for prevention of colonization and infection by typically antibiotic resistant pathogens that typically acquire antibiotic resistance. However, despite a multitude of early developments and publications there has been a long history of failures in clinical development of this type of vaccines (4–6). This review will set its emphasis on providing insight into the reasons that led to discontinuation of vaccine development programs and the consequences for clinical trial design.

## Target Pathogens for Vaccine-Based Approaches Against Nosocomial Bacterial Infections

Classical vaccine development has mainly focused on bacterial infections caused by toxin production (Tetanus, Diphtheria, Pertussis) or bacteria such as meningococci, pneumococci and *Mycobacterium tuberculosis* that cause severe, sometimes lethal infections and easily spread among the population. In the former category, disease is limited to the presence of toxins, e.g., it occurs upon infestation of Tetanus toxin in wounds or secretion of Diphtheria and Pertussis toxins by Corynebacteria and Bordetella species in the respiratory tract. The presence of toxin-neutralizing antibodies (induced by vaccination) mediates protection and can, thus, be quantified in international units (absolute correlate of protection) (7). It is further important to note that both these upper respiratory tract infections as well as the infections caused by meningococci, pneumococci, and *M. tuberculosis* are transmitted from human-to-human via droplets from nasal and respiratory secretions. Notably, the human is the main reservoir for transmission of these pathogens and, thus, vaccination has proven to be an efficient measure for protection on a population basis and containment of spread of these diseases.

In the context of antibiotic resistance, clinicians highlighted the importance of the ESKAPE pathogens, e.g., *Enterococcus faecium, Staphylococcus aureus, Klebsiella pneumoniae, Acinetobacter baumannii, Pseudomonas aeruginosa*, and *Enterobacter* species (8, 9). This acronym summarized the most frequently encountered pathogens in hospital-acquired bacterial infections ranging from wound infections and ventilator-associated pneumonia to sepsis. Nevertheless, other infections such as those caused by *Clostridioidales difficile* have increased in frequency and antibiotic resistance rates (10) and have, thus, been added to the list of nosocomial pathogens and potential bacterial targets for vaccine development. They are now frequently referred to as ESCAPE pathogens (*Enterococcus faecium, Staphylococcus aureus, C. difficile, Acinetobacter baumannii, Pseudomonas aeruginosa*, and *Enterobacteriaceae*).

## Characteristic Features of Nosocomial Bacterial Infections

Nosocomial infections are caused by bacterial pathogens either transmitted in the hospital environment or from commensals that were already present prior to hospitalization (endogenous infection). On an individual patient level, it is often cumbersome to follow up, which of these sources was causative, unless there is evidence for spread of a specific strain among patients. Moreover, the species causing hospital-acquired infections behave as facultative pathogens, indicating that they cause infections only in a subgroup of patients, under specific circumstances that are also hard to assess in the patient: a coincidence of transient (or chronic) immune suppression associated with age, co-morbidities and medical treatment, selection of resistant strains and dysbiosis caused by antibacterial therapies, transient (or chronic) disturbance of cutaneous and epithelial barriers, and possible displacement to other body areas (urinary tract infection or pneumonia caused by enteric bacteria). Lastly, the specific immune defense mechanisms and the ambiguous role of preexisting immune memory to microbiota are poorly understood.

## Expectations and Concerns With Vaccination Against Nosocomial Bacterial Pathogens

One of the major drivers for development of vaccines against nosocomial bacterial pathogens is that antibiotics are no longer effective in all patients. Notably, for most of the ESCAPE pathogens the reservoirs include zoonotic and environmental habitats such as animal husbandry and wastewater, where they are subject to continuous antibiotic selection pressure. It is, therefore, nearly impossible to eradicate these pathogens or revert their resistance by immunization programs in humans. More comprehensive One Health strategies are needed to reduce the antimicrobial resistance burden arising from these sources (11). Nevertheless, vaccine-mediated prevention of nosocomial infections in patients could reduce antibiotic usage and resistance development in hospitals.

Thus, the expectation is to prevent transmission and infection, avoid antibiotic therapy and reduce development of or revert antibiotic resistance [reviewed in (12)]. Two examples may highlight that along with other antibiotic stewardship measures vaccine-based prevention of infections could have the potential to reduce antibiotic usage and—at least transiently—positively influence resistance trends:

1. An indirect effect is postulated in relation to the seasonal influenza burden: reduced antibiotic usage due to vaccine-induced protection against influenza (13) could result in reduced rates of *C. difficile* infection, which shows seasonal co-incidence with influenza (14, 15). However, this intriguing hypothesis remains to be confirmed.
2. The introduction of pneumococcal conjugate vaccines re-shaped the epidemiological representation of pneumococcal serotypes. Initially, the reduction in infections with antibiotic resistant serotypes suggested that vaccines could reduce antimicrobial resistance. However, long-term analyses revealed that the beneficial effects on antibiotic susceptibility profiles could not be maintained after serotype replacement (16–19).

One repeated concern has been that eradication of specific commensals might negatively affect the resident microbiota composition and the local immune response. On the one hand, absence of a previously colonizing pathogen and concomitant loss of continuous exposure of the immune system to this pathogen could weaken immune defense and increase susceptibility for infection with this pathogen (20). On the other hand, manipulation of the microbiome creates niches for replacement by foreign strains or other species as exemplified for *S. aureus* (21, 22), which can potentially affect susceptibility to infection. It can only be speculated whether these effects might have contributed to the clinical failure of V710 (Merck), the only vaccine formulation, so far, that was specifically targeting nasal colonization with *S. aureus* (23). Dedicated research is needed to provide a better understanding of the complex interrelationships of microbiota and the immune system as well as to ensure safety of future developments.

## New Paradigms in Vaccine Development Targeting Nosocomial Bacterial Pathogens

One of the most obvious challenges for vaccine development is the high genetic diversity of strains typical of the commensal pathogens. The genetic heterogeneity translates into differences in chemical structure of variable proteins and polysaccharides and alters their immunogenicity. Immunological strain variability limits cross-reactivity of the immune response to variable surface proteins and polysaccharides and undermines vaccine-mediated cross-protection against strains not included in the vaccine design.

The lack of cross-protectivity has hindered the development of vaccines against several ESCAPE pathogens:

1. *P. aeruginosa* is an ubiquitously encountered environmental pathogen that favors humid environments. It can colonize the human mucosa in predisposed patients with altered or damaged epithelial barriers due to cystic fibrosis, ventilation (burn), wounds, and chemotherapy. Vaccines against *P. aeruginosa* were developed for three different target populations, e.g., cystic fibrosis, burn wounds and ventilator-associated pneumonia [reviewed in (5, 24)]. So far, the majority of vaccines that reached the clinical development stage targeted the lipopolysaccharide (LPS) and flagellar compounds with and without conjugation to protein carriers. These are highly immunogenic structures and antibodies against these compounds form part of the natural immune response in humans. Induction of LPS- and flagella-specific antibodies conferred protection in preclinical infection models. However, in the clinical trials, failure to prove efficacy was attributed to strain-dependent variability of LPS, Flagella and whole cell vaccines and insufficient coverage of the arsenal of different strains encountered in the patient population.
2. *K. pneumoniae* is a high-risk pathogen because it accumulates genetic resistance elements and easily spreads among patients, two features that favor the global spread of some highly virulent carbapenem-resistant strains. The species is characterized by the formation of a polysaccharide capsule, which acts as an important virulence factor. Early vaccines developments were, therefore, based on combinations of unconjugated and, later, conjugated capsular polysaccharide (CPS) antigens (5, 25). However, there are more than 70 serotypes with varying distribution worldwide. Although a multivalent vaccine covering 24 CPS was tested in a clinical trial (26). Although this vaccine was later tested in combination with 8-valent LPS vaccine against *P. aeruginosa* (27), development was later abandoned because it did not seem feasible to achieve protection against the multitude of different serotypes on a global level.

As an answer to the previous failures and the eminent clinical need, we have recently witnessed a change in paradigm: new vaccine developments no longer focus on broad coverage of strains but narrow the spectrum to individual, epidemiologically relevant strains known to harbor resistance to carbapenems. These developments include

1. Targeting of the capsule of *K. pneumoniae* based on a recently described semi-synthetic hexasaccharide-glycoconjugate (with CRM197 as protein carrier). The glyoconjugate was shown to induce antibodies with opsonophagocytic activity against carbapenem-resistant *K. pneumonia*e strains *in vitro* (28) and monoclonal antibodies derived thereof promoted protection against the *K. pneumoniae* ST258 strain *in vivo* (29).
2. Strain-specific LPS (O-glycan)-based developments of monoclonal antibodies targeting *K. pneumoniae* strains such as ST258 and *E. coli* strains ST131 (30– 32) have been developed to protect from epidemic strains with high transmission potential, antibiotic resistance and severe disease manifestation. Notably, serotype-specific vaccines have also been proposed for targeting O111 *E. coli* due to this serotype's high representation in toxin producing *E. coli* (EHEC, STEC, EPEC, and EAEC) (33, 34). To avoid toxicity in these vaccines O111 LPS was conjugated to carrier proteins.
3. Recent data further highlighted that small glycan motifs detected in a broad range of strains (and species) are natural antibody targets and could have the potential to serve as vaccine antigens (35–38).

Altogether, this development suggests that in light of global spread of antibiotic resistant strains exploring strain-specific vaccines may be worthwhile and abandoning the concept of broad strain coverage by vaccines may facilitate targeted vaccine development in the AMR field.

## The Immunological Mechanism of Action

The immunological mechanism of action of a vaccine usually refers to a known immune correlate of protection. The identification of this parameter requires investigation of the natural immune responses leading to the resolution of infection and vaccine-related immune protection. The currently licensed vaccines for prevention of bacterial infections either generate toxin-neutralizing antibodies, or enable clearance of bacteria via formation of bacterial immune complexes and subsequent phagocytosis. The studies sometimes use “immunological surrogates of protection” to correlate immunogenicity with protection and to provide an absolute quantifiable value for protection by measuring specific serum antibody titers and defining threshold levels (7). However, for bacterial pathogens causing nosocomial infections the correlates of protection remain unknown. Additionally, for most pathogens it is unclear whether the preexisting, natural immune response is protective.

For vaccine development it is of primary importance to understand, which type of immune response is needed to achieve the intended effect, e.g., prevention (or reduction) of colonization vs. prevention of infection and disease. However, there is a current lack of understanding of the immune response to most of the nosocomial bacterial pathogens.

Immunization with endogenous *S. aureus* and *E. coli* strains in parallel to the initiation of an antibiotic therapy prevented recolonization with these strains in mice, highlighting the efficacy of a systemic immune response in targeting these pathogens (39). However, at present it is unclear to which degree preformed natural immune responses are protective and whether Th2-dominated responses should considered as tolerogenic due to their failure to clear the commensal pathogens from the mucosal surfaces. It is, thus, very relevant to establish whether vaccine-mediated boosting of preexisting immune responses is sufficient for protective action of a vaccine or whether vaccines need to re-shape the immune response. While a Th2 response, which is linked to antibody production, may be effective in promoting protection against toxin-mediated diseases such as tetanus and diphtheria, this may be insufficient for infections where inflammatory T cell-mediated immunity (Th1/Th17) is required for immune defense. This could imply that *de novo* formation of immune memory or re-education of an established immune response by a vaccine could be required to induce protection.

There are three fundamental mechanisms responsible for vaccine-mediated protection:

1. Neutralization of toxins as drivers of disease: In the attempt to develop vaccines against nosocomial bacterial infections, many different strategies have been evaluated. Prominent examples for toxin-based vaccination strategies are vaccines targeting *C. difficile* toxins tcdA and tcdB to prevent manifestation of *C. difficile* infection (CDI). Additionally, some vaccine prototypes (and monoclonal antibodies) are targeting *S. aureus* toxins such as alpha toxin (hemolysin A), staphylococcal eneterotoxin B (SEB) and other secreted toxins such as leukocidins [reviewed in (4, 5, 40–42)]. The general assumption is that raising the level of neutralizing antibodies against toxins prevents invasive disease and lowers disease severity. It can further be speculated that immunization with *C. difficile* or *S. aureus* inactivated toxin antigens relies on a preexisting humoral immune response to the natural toxins and acts as a booster vaccination. Similarly, anti-toxin antibodies can be generated by immunization against toxins from toxigenic *E. coli* (43) while this approach is not feasible for protection against non-toxigenic *E. coli* such as extraintestinal *E. coli* harboring extended spectrum betalactamases (ESBL), which are often referred to as ExPEC.
2. Opsonophagocytosis of infecting pathogens: vaccines targeting bacterial surface molecules usually act by promoting opsonophagocytosis through antibodies and subsequent intracellular lysis of the bacterial pathogens. This mechanism is exploited by nearly all vaccine developments ranging from whole cells to formulations consisting of single or combined antigens. Multiple targets have been described for most ESCAPE pathogens [reviewed in (4, 5, 24, 41, 44)]. These include polysaccharides such as LPS, CPS, conserved glycan motifs and highly conserved immunogenic surface proteins such as outer membrane proteins (OMP). Nevertheless, only few vaccines targeting bacterial surface antigens from nosocomial bacterial pathogens have reached the stage of clinical development, among these mainly *S. aureus* vaccines (5, 40, 41, 45).
3. Shaping T cell immunity: the role of T cell-mediated immunity in defense against extracellular bacteria has been neglected although some of these pathogens, e.g., *A. baumanii* and *S. aureus*, reside intracellularly (46–50). Furthermore, different types of T cell responses might be required depending on the body compartment (51, 52). For example, Th17 responses are relevant in the skin and mucosal surfaces in defense against *S. aureus* (53, 54), and have also been found to be important for clearance of ESCAPE pathogens such as *Klebsiella* spp., *P. aeruginosa* and *A. baumanii* (55–64). Thus, vaccine formulations targeting colonizing pathogens might need to be optimized to induce Th17 responses (24, 58, 65). However, due to the paucity of data, further investigation is needed to identify the specific T cell responses required for protection, and understand, whether vaccine-induced long-term immunity is preferable to acute induction of immune memory in short interval to infection.

**Figure 1:**
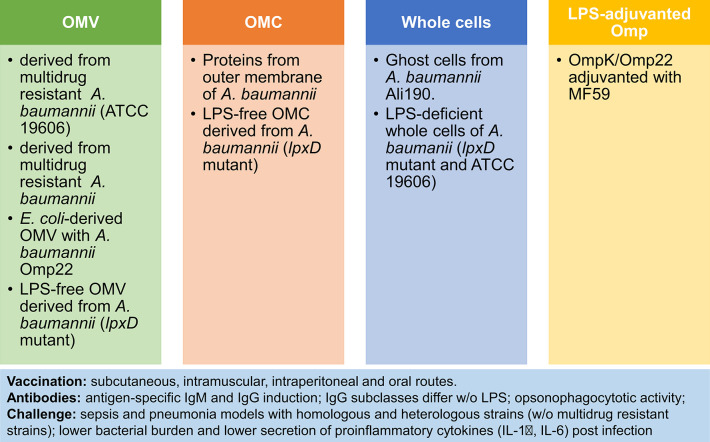
OMV-based and related approaches to vaccination against *A. baumannii*. Four immunization strategies have been tested in preclinical models of sepsis and pneumonia: (1) Outer Membrane Vesicles (OMV) (70–73); (2) Outer Membrane Complex (OMC) (74, 75); (3) Whole cells (76, 77); (4) LPS-adjuvanted Omp (78). Comparison of vaccines reveals higher potency of LPS-containing vaccines. Notably, the vaccine response is characterized by antibody induction and reduced pro-inflammatory responses after challenge, next to improved survival and lower bacterial burden post-infection.

Although initially, the LPS content in outer membrane vesicles (OMV) was regarded a safety issue, OMV have gained acceptance as vaccine components after licensing of the MenB vaccine Bexsero (66). These vesicles are physiologically released by Gram negative bacteria and embed several surface antigens in a lipophilic vesicular structure. They resemble bacterial cells because they combine protein antigens, polysaccharides and molecules with innate immune stimulatory properties such as LPS, lipopeptides, lipoteichoic acid and peptidoglycan. Next to the induction of opsonizing antibodies, OMV trigger Th1/Th17 cell responses. For ESCAPE pathogens several OMV-based approaches to vaccination have been evaluated at the preclinical stage (66). The diversity of these approaches to OMV-based or related vaccines can be exemplified by summarizing those evaluated for *A. baumannii* (summarized in Figure 1) [reviewed in (67–69)].

## Consequences for Design of Efficacy Studies

One major factor complicating vaccine trials for nosocomial pathogens is that the patients affected are elderly individuals. They suffer of co-morbidities and are at risk for immune suppression through both medical intervention and immunosenescence. Their immune status is difficult to assess with current diagnostic methods but it predisposes for infections and antibiotic therapy. A study on *A. baumannii* pneumonia in aged mice illustrates that mortality increases with age, while efficacies of treatment, both antibiotics and vaccination, decrease because both rely on functional immune responses (79). Similarly, the human vaccine response is compromised by the immunodeficiencies caused by aging of the immune system [reviewed in (80, 81)].

The clinical development of vaccines for prevention of hospital-acquired infections is linked to a history of failures. Today, the major challenges are well-understood and variables such as immune deficiency due to aging of the immune system are being taken into account and reflected by new vaccine formulations (82, 83). Nevertheless, it remains difficult for trial design to predict some known factors that seem out of control and hit rates are often lower than expected (84). As summarized in Table 1 the main enemy in trial design for this type of vaccines is *time*.

**Table 1:** Challenges in clinical trial design for vaccine development to prevent hospital-acquired infections.

**Study parameter**	**Problem statement**	**Implications and potential solutions**
Patient recruitment	It is difficult to define the patients at risk long beforehand. Hospital-acquired infections are typically associated with unplanned events such as cardiac surgery or ICU ventilation; adding to the difficulty, patients clearly at risk are often no longer able to sign informed consent	Since early involvement of future patients is key to success, multi-stakeholder cooperation is need and a fallback on nation-wide registries and cohorts could be very valuable
Diagnostics	Precise methodology for distinguishing infection from colonization and for detection of potential co-infections as important variables is frequently not in place and, thus, delays diagnosis or its accuracy. This further has impact on the precision of inclusion and exclusion criteria and clinical endpoints. Additionally, diagnosis of immune status and microbiome composition are not routinely collected	Diagnostic method development should be fostered to make rapid, comprehensive and precise diagnostics available. The value of immune status and microbiome assessment needs to be evaluated
Vaccination schedule	Late recruitment bears the risk that time between vaccination and disease manifestation is too short for establishment of stable immune memory and required booster vaccination	Vaccination schedules will vary depending on the proposed immunological mechanism of action. Boostering of an existing immune response is different from reshaping or *de novo* formation of an immune response. Induction of T cell immunity vs. antibody responses will require different approaches
Unpredictability of infection	Infection is unpredictable in regards to the time point of disease manifestation and the patients affected in the cohort. Manifestation of infection might not fall within the duration of the study. Examples highlighting this issue are bloodstream and prosthesis infections where infection rates vary strongly	Pre-established clinical trial networks with the flexibility to recruit patients from many different trial sites may have a great advantage to recruit a sufficiently high amount of subjects
Choice of clinical trial sites	Epidemiology is subject to change. Global spread of strains with antibiotic resistance and high transmission potential and environmental fitness changes the representation of strains over longer periods. Even more important for a clinical study, the local epidemiology varies. These changes are sometimes hard to track because they depend on multiple factors, e.g., regional representation of strains, infection control measures and antibiotic regimens. Consequently, hit rates at a study site can be unexpectedly low, thus diminishing the statistical power of the studies. One prominent example is that incidence of ventilator-associated pneumonia with *P. aeruginosa* on ICUs has declined, which might be attributed to the introduction of more rigorous infection control measures and standardized procedures	Pre-established clinical trial networks with well-characterized sites and information on local epidemiology and updates on changes in routine antibiotic regimens and infection control measures may be detrimental in commissioning of suitable sites and recruitment of study subjects. The network structure could facilitate and speed up the process
Clinical endpoints	Clinical endpoints such as survival or pneumonia on an ICU are often too broad and ambitious in their scope	Clinical endpoints should be based on the precise diagnosis and prevention of an infection with a specific target pathogen and co-infections excluded

### Controlled Human Infection Models

One alternative to obtain efficacy data is to develop controlled human infection models (CHIM) and use these models for evaluation of vaccines (Human Challenge Trials, HCT) (85). Lately, this type of studies has gained more importance for proof-of-concept studies, licensing and prequalification of vaccines. In particular, indications where epidemiology of disease does not allow the timely execution of the clinical efficacy trials these studies have become relevant for decision making of regulators and developers. However, there are significant limitations to this type of trials that need to be considered:

- The safety of the study participants is the most important requirement: infection has to be controlled, e.g., appropriate treatment has to be available and clearance of the pathogen guaranteed.
- Clinical endpoints must be clinically relevant and reflect the natural course of disease
- The choice of infectious dose, the virulence of the challenge pathogen and severity of disease manifestation should resemble natural course of infection without compromising the safety of the study participant.
- The number of study participants is typically small.
- 100% colonization rates or 100% infection rates may be required to obtain significant results but may be difficult to achieve in practice.
- The studies have limited value if the challenge agent does not reflect the epidemiologically relevant spectrum of strains encountered in real life.
- Standardization of procedures (administration and manufacturing of the challenge agent, the challenge agent itself, disease severity scores, primary and secondary endpoints) is essential for comparability of the studies and vaccines but often not in place.

In vaccine development HCT studies are well-established for many indications. For bacteria, CHIM have mainly been used in the development of vaccines against enteric pathogens among them enterotoxigenic *E. coli* (ETEC), *Shigella* spp. and *V. cholera*. The learnings included that HCT results do not predict vaccine effectiveness in field studies (86). Substantial efforts for standardization have been made to achieve high quality and reliability of results in ETEC and Shigella studies (87–89).

To date, no human challenge models have been published for evaluation of vaccines for hospital-acquired bacterial infections. A few studies addressed the colonization potential of *S. aureus* (ST398) (90) and non-toxigenic *C. difficile* (91, 92). Although proof-of-concept studies could be supportive, accounting for the heterogeneity of strains, patients (immune status, dysbiosis, barrier function of epithelia) and disease course would not be feasible in this setting. This questions the relevance of data collected in HCT and, in this context, limits utility to CHIM studies related to understanding of the role of the immune response and microbiota in prevention and resolution of infection and, potentially, very specific research questions such as decolonization of patients colonized with multidrug resistant pathogens.

## Synergies and Redundancies of Microbiological and Immunological Defense Mechanisms

Another aspect that influences clinical trials to a so far unknown extent is that host defense is prepared to fight infections with two different strategies: next to the host immune system reconstitution and resilience of the host microbiome limits spread and promotes clearance of the infecting pathogen (93). Although many studies have addressed the interplay of both of these systems in the healthy setting the synergies and redundancies are is not well-understood in the context of an infection and is even less controllable in the clinical trial setting. These functionally distinct, but redundant, immunological and microbiological defense mechanisms have evolved to secure protection from infection. Their presence, absence or resurgence (resilience of the microbiome, recovery of the immune response) could superimpose vaccine-mediated resolution of infection resulting in failure to demonstrate efficacy in the clinical trial.

Recent reports indicate that the microbiota influence the vaccine response. From studies in low income countries we learned that many factors including the microbiota composition can influence the response to oral vaccination (94). It is further well-known that the microbiota are essential for the development of the intestinal immune system. Among them, segmented filamentous bacteria have been associated with recruitment of Th17 cells to the gut (95). Furthermore, the abundance of specific microbiota correlated with higher immunogenicity, e.g., Actinobacteria, in particular the Bifidobacteria genus, were found to increase immunogenicity of OPV in Bangladesh and low representation of Bacteroidetes and high abundance of Bacilli (*Streptococcus bovis*) correlated with better vaccine response and seroconversion in rural Ghana (96, 97). Additionally, treatment of mice with antibiotics reduced the polio replication and infectivity in mice, a phenomenon well in line with previous findings that the microbiota enhance uptake and replication of enteric viruses (98, 99). However, this was not observed in a trial using azithromycin in children (100). Similarly to Polio vaccine virus, shedding of the Rota virus was increased if subjects were treated with antibiotics before vaccination but no differences in IgA levels were observed (101, 102). By contrast, pretreatment with antibiotics reduced the neutralizing antibody response to influenza vaccination, indicating that systemic immune responses are also affected by the lack of microbiota (103).

The pathogens causing nosocomial infections usually reside on the mucosal surfaces. On the one hand, microbiota colonizing the mucosa constantly stimulate epithelia and innate immune cells, trigger the production of antimicrobial peptides and IgA and shape the T and B cell repertoires [reviewed in (104)]. On the other hand, these factors play an important role in regulating the quantity and composition of the local microbiome as well as the defense against invading pathogens (105, 106). Notably, the presence of IgA protects mice from DSS colitis and polymicrobial sepsis (107, 108).

Recent evidence suggests that administration of antibiotics leads to loss of IgA secretion. This was demonstrated in the respiratory tract where IgA-deficiency was associated with increased susceptibility to infection with *P. aeruginosa* (109). Similarly, in a humanized mouse model of IgA nephropathy treatment with antibiotics prevented renal deposition of IgA complexes, which was proposed to be due to a reduction of the intestinal microbiota and the concomitant loss of the microbiota-specific IgA (110).

## Microbiological and Immunological Susceptibility to *C. difficile* Infection

**Figure 2:**
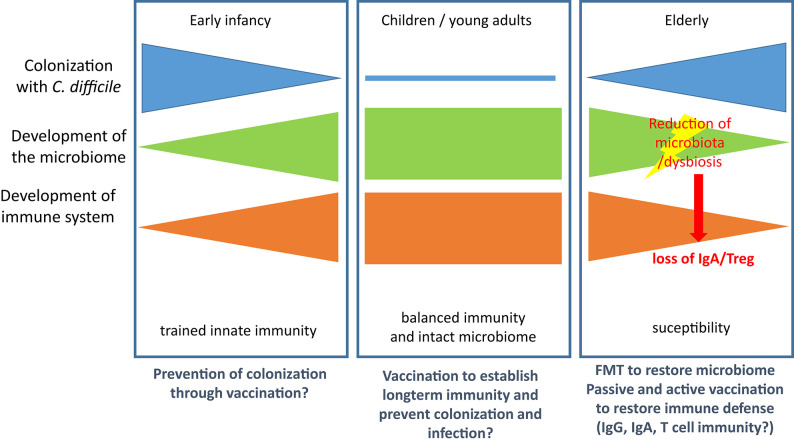
Microbiological and immunological defense against CDI. **(Left)**: Colonization is established in early infancy but regresses in the first year of life with both the maturation of the immune system and the development of the full microbiome. **(Middle)**: In children and young adults the immune system is balanced and the microbiome intact. Both factors control growth of *C. diffcile* and toxin production. **(Right)**: In the elderly population the microbiome and the immune system are both subject to age-related changes, which leads to increased susceptibility for CDI. Treatment with antibiotics results in reduction of microbiota and dysbiosis and enables growth of *C. difficile*. Loss of microbiota-mediated stimulation of immune cells leads to loss of IgA secretion and Treg and thereby facilitates CDI and the associated inflammatory processes. Therapeutic options are provided below the panels.

Colonization with *C. difficile* occurs at very early age and is detectable in 80% of newborns in their first month of life. It decreases to colonization rates of 3% (comparable with adults) by the end of the first year of life (111). Thus, trained innate immunity is probably established during this early phase of life and shapes the tolerogenic immune response to the colonizing pathogen. As for other hospital-acquired infections in the elderly the risk for CDI is increased, which might be attributed to both immunosenescence (112) and age-related changes in the gut microbiota (113). However, the relative contribution to susceptibility remains ill-defined for both factors and is complicated by the reciprocal regulation of intestinal microbiota and mucosal immunity. Figure 2 summarizes the major events contributing to CDI susceptibility.

Based on the findings described above it can be speculated that an antibiotic-induced IgA-deficiency could account for susceptibility to *C. difficile* infection (CDI) (114). High titers of toxin (TcdA and TcdB)-specific antibodies, in particular IgA in serum and feces, correlate with protection against CDI, while low titers or absence of toxin-specific IgG and IgA were found in patients with acute or recurrent CDI and in non-colonized individuals (115–118). These data indicated that patients with transient deficiency in IgA might be more susceptible for infection. Well in line with this observation Bezlotoxumab, a monoclonal antibodies directed against TcdB, prevented recurrence of CDI, highlighting the value of antibody-mediated toxin neutralization (119, 120).

The role of cellular immunity in CDI is less well-understood. Notably, HIV+ individuals with low CD4+ T cell counts and homozygotes with a Q223R mutation in the leptin receptor, which abrogates synthesis of IL-23, a cytokine that induces formation of Th17 cells, have an increased risk for CDI (121, 122). It is further known that high levels of T cell-derived cytokines (IFNγ and IL-5) in peripheral blood correlate with less severe disease manifestation (123). IL-23 is elevated in feces and intestinal biopsies of CDI patients (124, 125) and patients with recurrent CDI display increased numbers of Th1 and Th17 cells in peripheral blood (126). The role of these inflammatory T cell subsets is, however, controversial. Recent data highlight their contribution to immune pathology of CDI rather than a protective role (127). It has further been suggested that microbiota induce Treg and regulate the balance between Treg and Th17 cells (128). In analogy to IgA, the reduction of microbiota by antibiotics could, thus, increase susceptibility to CDI and, in particular, contribute to inflammation and immune pathology by relaxing Treg-mediated suppression and allowing increased formation of Th17 cells.

Although these data argue for an important contribution of both T and B lymphocytes to the immune response in CDI, Leslie et al. recently demonstrated in a mouse model that clearance of *C. difficile* due to resilience of the microbiome occurs in the absence of adaptive immune responses (129), arguing for a non-essential role of immune defense in this context. Furthermore, colonization with non-toxigenic strains can prevent colonization with toxigenic strains of *C. difficile* and, thus, prevent disease, which was demonstrated in the hamster and in recurrent human CDI (92, 130). Similarly, fecal microbiota transplantation (FMT) has become a success story in treatment of CDI (131). Nevertheless, recent studies indicate that non-immune, soluble factors such as such as butyrate and bile salts or bacteriophages might play an underestimated role in reconstitution of the microbiota after CDI (131–134). However, the model developed by Leslie et al. did not consider the effects of aging of the microbiome (113). It is, therefore, likely that age-related changes in the immune system and the microbiota facilitate colonization with *C. diffcile* and development of CDI and that regeneration of one or both systems drives resolution if infection (Figure 2).

## Conclusions and Perspectives

**Figure 3:**
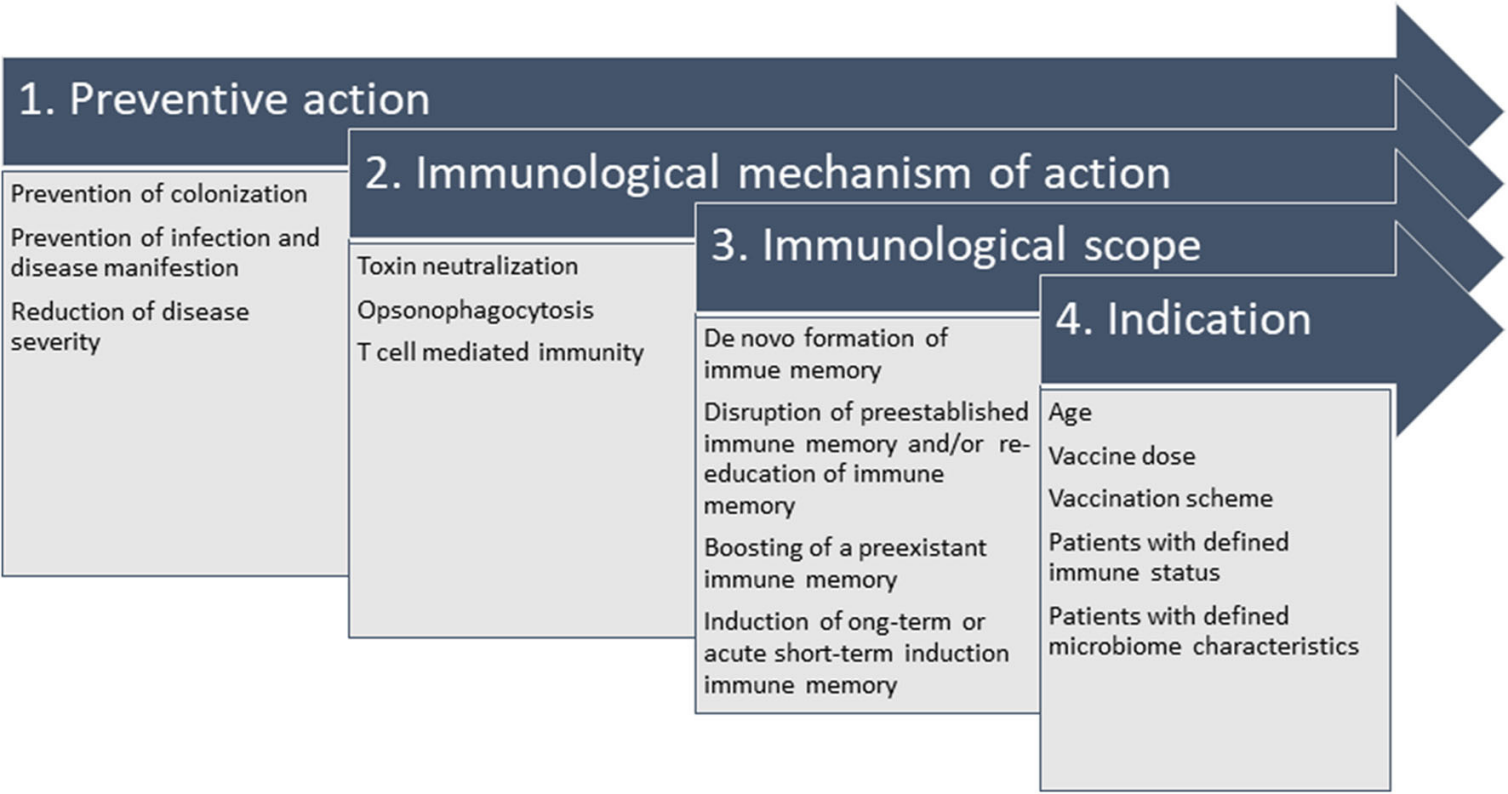
Interconnection of parameters relevant to design the vaccination strategy against nosocomial bacterial pathogens. It is crucial to define the desired preventive action (1), which defines the immunological mechanism of action (2) when set in context with the available knowledge of pathophysiology, e.g., intra- and extracellular survival, correlate of protection (or a surrogate, if unknown). In the specific case of hospital-acquired infections, colonization in youth or in elderly patients at risk precedes infection and vaccine design needs to take into account that natural immune responses to colonizing pathogens might not be protective. Thus, they might need refurbishing with the immunological scope (3) of strengthening preexistent immune responses (“booster”), establishing long-term protective immune memory or promoting immunity by acute intervention. All considerations generate the indication (4), e.g., all details on administration (e.g., vaccination scheme and dosage) and indication for defined patient populations (age indication, immune status, microbiome).

These multiple findings highlight the complexity behind the endeavor to develop vaccines for nosocomial bacterial infections. The heterogeneity of patients, bacterial strains, disease course and hospital epidemiology have complicated vaccine development in this area and have led to discontinuation of vaccine development programs. However, the power of vaccine prevention has been demonstrated in many occasions. Thus, re-thinking the strategies may be warranted but failures should not be accepted without further refurbishing on available and new scientific data. For new developments, the definition of pathogen-specific clinical endpoints and the suitability of the immunological mechanism of action are key to success. Figure 3 summarizes the interconnection of the relevant parameters, which influence the trail design and final indication.

For vaccines targeting *K. pneumoniae* and *E. coli* there is a trend to focus on epidemiologically relevant strains. A next step could be the development vaccine formulations that trigger specific, protective T cell responses such as Th17 cells in the mucosa. However, future research will need to define the type of T cell responses required and the route of immunization needed to establish protection in different body compartments. Next to the quality of the T cell response it will be relevant to understand the optimal time point for vaccination: (1) whether it is preferable that vaccines build on establishment of long-term immunity, or, (2) whether immunization at short temporal distance to onset of infection is favorable because acute formation of T cell immunity is more effective in promoting protection against infection, although the vaccine response in the latter case may be short-lived.

Despite the higher cost, a more detailed characterization of individual patients and patient subpopulations within the trial population seems warranted to ensure that the results obtained in the clinical trials are meaningful. One further step could be to identify the patient population that benefits from vaccination. Pro- and retrospective analyses of trial data on subject level could help to define the characteristics of these patient collectives and improve stratification in future trials. This concept is not new but well in line with the current understanding of personalized medicine and individualized treatment concepts that have recently been introduced to the field of infectious diseases, antibiotics and vaccination (135–137). In light of the interdependency of immune status and microbiome resilience, the influence of these factors on clinical trial success needs to be investigated more thoroughly.

## Author Contributions

IB-D developed the concept, performed the analyses, and wrote the paper.

## Conflict of Interest

The author declares that the research was conducted in the absence of any commercial or financial relationships that could be construed as a potential conflict of interest.
